# Peer review of "Investigating the IgM and IgG B Cell Receptor Repertoires and Expression of Ultralong Complementarity Determining Region 3 in Colostrum and Blood from Holstein-Friesian Cows at Calving"

_animals, 2024, doi:10.3390/ani14192841_

Round 1

Reviewer 1 Report

Comments and Suggestions for Authors

Investigating the IgM and IgG B cell Receptor Repertoires and Expression of Ultralong Complementarity Determining Region 3 in Colostrum and Blood from Holstein-Friesian Cows at Calving

Background and overall summary

Adoptive transfer of maternal antibodies to newborn calves is considered essential during the early stages of immunity and without placental transfer, the calves rely on receiving these antibodies through their nutrient-rich first milk, or colostrum. Colostrum contains a mixture of immunoglobulins (IgG, IgM and IgA) and the calf relies on this passive immunity process for protection against infection and disease until its own immune system is mature enough to take over. In addition to this, high levels of maternal IgG need to be present in the colostrum to ensure adequate protection.

[Ref. Skirving, R.; Bottema,C.D.K.; Laven, R.; Hue, D.T.;Petrovski, K.R. Incidence of Inadequate Transfer of Passive Immunity in Dairy Heifer Calves inSouth Australia. Animals 2022, 12,2912. https://doi.org/10.3390/ani12212912.]

However, the protection offered by passive immunity wanes quickly, typically within the first 1 or 2 days of life leaving a window of high-risk before active immunity is established. This is well documented.

Cattle are unique in that they possess antibodies with ultralong CDR3. The unique structure of these ultralong CDR3 is thought to increase diversity, allowing the antibody to recognise and bind to a much broader range of antigens. This increased antigen recognition is thought to enhance the cattle’s immune response. Here the authors hypothesis a link between importance of passive immunity with an increased presence of ultralong CDR3 antibodies in colostrum. Much of their reported work is based on a recent methods publication using the Long-read Oxford Nanopore MinION Platform.

Here the authors report for the first-time direct comparison of relative percentages of ultralong CDR3 in blood and colostrum in dams at the point of calving. They report significantly higher percentages of IgM and IgG B cells with ultralong CDR3 in colostrum compared with blood and indicate that this may be linked to a potential protective role of the neonate. Indeed, this makes sense, and it would be very interesting to see this followed up with a neonatal study. However, I have some doubts about the filtering methods used, which form the basis of the data analysis of the IgM and IgG BCR repertoires used throughout this study, and the relative percentages quoted. I would recommend that they run some of their sequences through an alternative diagnostic software tool (as mentioned below) to verify their data. Also, as I mention below in more detail, there is confusion between the tables in terms of each cow’s data. I suggest removing the ‘cow’ that only has colostrum/no blood sequences to remove confusion and provide the reader with more confidence that the blood and colostrum sequences are directly related to each cow.

Introduction

Quoted cell numbers of 5x105 cells/ml in colostrum with approximately 3.5% (1.75x104/ml) of which are B cells. Perhaps include an estimation for blood? For instance, 20-30% B cells (CD40/IgL+).

Suggestion for introduction - similar areas of research on cattle ultralong antibodies not included:

[J. D.  Clarke, A.  Douangamath, H.  Mikolajek, M.  Bonnet-Di Placido, J.  Ren, E. E.  Fry, D. I.  Stuart, J. A.  Hammond and R. J.  Owens. The impact of exchanging the light and heavy chains on the structures of bovine ultralong antibodies. Acta Crystallographica Section F 2024;80(7):154-163. https://doi.org/10.1107/S2053230X2400606X]

[Patrick Miqueu, Marina Guillet, Nicolas Degauque, Jean-Christophe Doré, Jean-Paul Soulillou, Sophie Brouard. Statistical analysis of CDR3 length distributions for the assessment of T and B cell repertoire biases. Molecular Immunology. Volume 44, Issue 6, 2007. Pages 1057-1064. ISSN 0161-5890. https://doi.org/10.1016/j.molimm.2006.06.026].

Materials and Method

2.3 Line 150. Mentions the differences in B cell population frequency between blood and colostrum – would be good to quote actual figures, either here or in the Introduction (as highlighted above).

2.5 Line 219. “Colostrum and blood samples of both isotypes from the same animal were run on the same MinION Mk1C flow cell (Oxford Nanopore Technologies, catalog# R10.4.1) and allowed to run for ~72 hours.” This is where the data needs clarification as this is not evident in the data tables (see below).

Figure 1. Sequences look like they are displayed in Word document or similar, may want to think about using software with annotation tools such as Mega, SnapGene, etc.

The IgM and IgG filtering process for the sequences is removing a lot! What was the starting read length? I used this data to align these sequences against our primers, the V primers align with all functional V gene segments of the reference genome and the J primers aligned, but the J is very upstream. Have you considered using IgMAT instead of the RSLB filtering method? IgMAT is a freely available diagnostic tool for exploring antibody repertoires

[Dorey-Robinson, D., Maccari, G. & Hammond, J.A. IgMAT: immunoglobulin sequence multi-species annotation tool for any species including those with incomplete antibody annotation or unusual characteristics. BMC Bioinformatics 24, 491 (2023). https://doi.org/10.1186/s12859-023-05624-2].

In table 1; blood IgM sequences using RSLB filtering and IMGT. Losing greater than 50% of raw reads using this filtering methods, but then only one-third decrease when looking at the ultralongs to productive protein and this is somewhat surprising as I would expect this to be closer to two-thirds. They also refer to IMGT High V-Quest filtering bioinformatic tool but then do not discuss analysis in detail in the paper. In table 3: blood IgG, they see similar numbers, 60% removal of raw reads and one-third decrease to productive protein. Likewise, table 4: colostral IgM has similar values of 50% and one-third. So at least this is consistent. But when looking at the average SEM from each table, this is much higher in the colostrum compared with blood and this is not discussed.

Inconsistencies in data reporting for Cows 1 to 8. When I tried to replicate the data in Figure 5: correlation of IgM ultralongs in colostrum and blood there was no correlation when I used Cows 1 to 7; I could only replicate Figure 5 when I removed Cow 8. Then I noticed a discrepancy in the ‘parity’ column on the tables. In Tables 4 and 5, Cow 1 has a parity of 3 and Cow 8 has a parity of 4. However, in Tables 1 and 3, Cow 1 has a parity of 3 and Cow 8 is not included. In Table 6, Cow 1 has a parity of 4 and Cow 8 has a parity of 1. This is making it impossible to directly compare blood against colostrum for each cow.

Discussion

Line 590. “There are currently no estimates of the percentage of colostral B cells expressing ultralong CDR3s, but the estimates from this study were higher than what is currently found in the literature regarding the percentage of B cells in blood with ultralong CDR3s.” I am in agreement with this statement, and I suspect part of this may be due to the MACS method of isolating IgG and IgM B cells from blood and the analysis tools used as mentioned above in my comments.

Line 612. The discussion would benefit from more-in depth comparison of IMGT versus RSLB analysis.

The paper is well written and informative and provides an interesting insight into the potential role of ultralong CDR3 antibodies in enhancing neonatal protection in the first few days of life. 

Author Response

Response to Reviewer 1 (Authors):

We would like to thank reviewer 1 for an in-depth review of our manuscript. We appreciate the identification of inconsistencies in our tables and bringing IgMAT and other important literature to our attention. We have addressed and rectified all concerns in the sections below.

Background and overall summary (Reviewer)

Adoptive transfer of maternal antibodies to newborn calves is considered essential during the early stages of immunity and without placental transfer, the calves rely on receiving these antibodies through their nutrient-rich first milk, or colostrum. Colostrum contains a mixture of immunoglobulins (IgG, IgM and IgA) and the calf relies on this passive immunity process for protection against infection and disease until its own immune system is mature enough to take over. In addition to this, high levels of maternal IgG need to be present in the colostrum to ensure adequate protection.

[Ref. Skirving, R.; Bottema,C.D.K.; Laven, R.; Hue, D.T.;Petrovski, K.R. Incidence of Inadequate Transfer of Passive Immunity in Dairy Heifer Calves inSouth Australia. Animals 2022, 12,2912. https://doi.org/10.3390/ani12212912]

However, the protection offered by passive immunity wanes quickly, typically within the first 1 or 2 days of life leaving a window of high-risk before active immunity is established. This is well documented.

Cattle are unique in that they possess antibodies with ultralong CDR3. The unique structure of these ultralong CDR3 is thought to increase diversity, allowing the antibody to recognise and bind to a much broader range of antigens. This increased antigen recognition is thought to enhance the cattle’s immune response. Here the authors hypothesis a link between importance of passive immunity with an increased presence of ultralong CDR3 antibodies in colostrum. Much of their reported work is based on a recent methods publication using the Long-read Oxford Nanopore MinION Platform.

Here the authors report for the first-time direct comparison of relative percentages of ultralong CDR3 in blood and colostrum in dams at the point of calving. They report significantly higher percentages of IgM and IgG B cells with ultralong CDR3 in colostrum compared with blood and indicate that this may be linked to a potential protective role of the neonate. Indeed, this makes sense, and it would be very interesting to see this followed up with a neonatal study.

However, I have some doubts about the filtering methods used, which form the basis of the data analysis of the IgM and IgG BCR repertoires used throughout this study, and the relative percentages quoted. I would recommend that they run some of their sequences through an alternative diagnostic software tool (as mentioned below) to verify their data.

Also, as I mention below in more detail, there is confusion between the tables in terms of each cow’s data. I suggest removing the ‘cow’ that only has colostrum/no blood sequences to remove confusion and provide the reader with more confidence that the blood and colostrum sequences are directly related to each cow.

Author response:

Thank you for providing an in-depth review of the manuscript. The main points for revision including the filtering method and the usage of IgMAT have been addressed below in the response to reviewers. The data regarding the 8 cows has been clarified and the cow with only a colostrum sample has been removed from the study.

Introduction

Comment 1: Quoted cell numbers of 5x10cells/ml in colostrum with approximately 3.5% (1.75x104/ml) of which are B cells. Perhaps include an estimation for blood? For instance, 20-30% B cells (CD40/IgL+).

Author response:

Thank you for the suggestion. We have included an estimation of the concentration of B cells in blood. Line 56 to 57.

Comment 2: Suggestion for introduction - similar areas of research on cattle ultralong antibodies not included:

[J. D.  Clarke, A.  Douangamath, H.  Mikolajek, M.  Bonnet-Di Placido, J.  Ren, E. E.  Fry, D. I.  Stuart, J. A.  Hammond and R. J.  Owens. The impact of exchanging the light and heavy chains on the structures of bovine ultralong antibodies. Acta Crystallographica Section F 2024;80(7):154-163. https://doi.org/10.1107/S2053230X2400606X]

[Patrick Miqueu, Marina Guillet, Nicolas Degauque, Jean-Christophe Doré, Jean-Paul Soulillou, Sophie Brouard. Statistical analysis of CDR3 length distributions for the assessment of T and B cell repertoire biases. Molecular Immunology. Volume 44, Issue 6, 2007. Pages 1057-1064. ISSN 0161-5890. https://doi.org/10.1016/j.molimm.2006.06.026 ].

Author response:

Thank you for sharing this literature. We have included the reference by Clarke et al., 2024 to our introduction. Line 95 to 96.

Materials and Method

Comment 3: Line 150. Mentions the differences in B cell population frequency between blood and colostrum – would be good to quote actual figures, either here or in the Introduction (as highlighted above).

Author response:

We have included the difference in B cell population from the literature, as we did in the introduction. Since colostral cells were not MACs sorted, it is not possible to quote the percentage of B cells in colostrum from this study. Line 137 to 138.

Comment 4: Line 219. “Colostrum and blood samples of both isotypes from the same animal were run on the same MinION Mk1C flow cell (Oxford Nanopore Technologies, catalog# R10.4.1) and allowed to run for ~72 hours.” This is where the data needs clarification as this is not evident in the data tables (see below).

Author response:

The errors in the table have been rectified. Each colostrum sample and blood sample from an individual were run on the same flow cell. We appreciate your diligence in reviewing our tables. Line 236 to 238. See Table 1 through 6.

Comment 5: Figure 1. Sequences look like they are displayed in Word document or similar, may want to think about using software with annotation tools such as Mega, SnapGene, etc. The IgM and IgG filtering process for the sequences is removing a lot! What was the starting read length? I used this data to align these sequences against our primers, the V primers align with all functional V gene segments of the reference genome and the J primers aligned, but the J is very upstream. Have you considered using IgMAT instead of the RSLB filtering method? IgMAT is a freely available diagnostic tool for exploring antibody repertoires. [Dorey-Robinson, D., Maccari, G. & Hammond, J.A. IgMAT: immunoglobulin sequence multi-species annotation tool for any species including those with incomplete antibody annotation or unusual characteristics. BMC Bioinformatics 24, 491 (2023). https://doi.org/10.1186/s12859-023-05624-2].

Author response:

We have created a better visual annotation of Figure 1 where the primers on the heavy chain for IgM and IgG are located. It was of critical importance to document isotype for our study and to document these sequences through RSLB filtering for our primers. See Figure 1.

The maximum length of the raw fastq files for all samples of IgM ranged from 9,172 to 83,192 nt and for IgG from 9,527 to 68,276 nt. Sequences were then filtered in the initial quality filtering steps of RSLB to remove IgM sequences longer than 1200 nt and IgG sequences longer than 800 nt. These extremely long sequences seen in the raw fastq files make up a very small percent of the total sequences and it was determined by manual curation that these extremely long sequences contain artefacts from ONT sequencing. After filtering by length, quality score, presence of the forward primer (V region), presence of the reverse primer (Isotype IgM or IgG), and presence of a pre- and post-CDR3 (Framework 3 and framework 4). The filtering steps decrease the number of sequences by ~50%. The filtering strategy removes artefacts that occur due to ONT long read sequencing, but also accommodates minor variations and maximizes the capture of ultralong CDR3 sequences, validating them for downstream analysis.

We used previously published primers for the constant region of the heavy chain of IgM (Ma et al., 2016) and IgG (Saini et al., 199). We did not target the J gene specifically. The reverse IgM primer is located much further downstream. This primer was previously used by Ma et al., 2016. The IgG reverse primer is located much closer to the J gene than our IgM primer. We initially sought an unbiased IgG HC primer further downstream, but other downstream HC IgG primers could not be accommodated by the PCR protocol. The current reverse IgG primer (Saini et al., 199) has also been successfully used by other groups. Based on the distribution of CDR3 length and the analysis from IMGT and RSLB filtering the J gene is located in the proper position post-CDR3.

Thank you for recommending IgMAT. We have not utilized IgMAT for our data, but it is an analysis we will complete and use to further validate our filtering method in the future. Currently we have data from cows and their calves. There are samples tracking calves from D0 (pre-colostrum) to approximately 10 months of age. Based on our preliminary analysis of the neonatal B cell repertoires compared to that of mature cattle, we anticipate that multiple different data training sets would be necessary in order to optimize the utility of IgMAT. The IgMAT analysis may prove valuable for this data set and we are excited to explore this tool further. We validated our search terms against IMGT and performed manual curation of nucleotide mismatches. We also compared the RSLB filtering method with IMGT in our methods paper.

Altvater-Hughes, T. E., Hodgins, H. P., Hodgins, D. C., Gallo, N. B., Chalmers, G. I., Ricker, N. D., & Mallard, B. A. (2024). Estimates of Sequences with Ultralong and Short CDR3s in the Bovine IgM B Cell Receptor Repertoire Using the Long-read Oxford Nanopore MinION Platform. ImmunoHorizons8(9), 635-651. https://doi.org/10.4049/immunohorizons.2400050

Comment 6: In table 1; blood IgM sequences using RSLB filtering and IMGT. Losing greater than 50% of raw reads using this filtering methods, but then only one-third decrease when looking at the ultralongs to productive protein and this is somewhat surprising as I would expect this to be closer to two-thirds. They also refer to IMGT High V-Quest filtering bioinformatic tool but then do not discuss analysis in detail in the paper.

Author response:

Whether we used refined stepwise-literature based filtering method (RSLB) or IMGT there was removal of approximately 50% of sequences, which may be due to the sequencing platform itself. The RSLB filtering method helps to reduce artefacts and helps to validate the sequences that make it through to the final analysis. Additionally, one third of the total sequences (on average, 32.65% of total sequences) is what was recovered as productive sequences. When translating sequences from RSLB filtered DNA to productive protein, on average, there was a loss of 21.20% sequences, which is similar to sequence loss in previous studies [Oyola et al., 2021; and Larsen et al., 2012]. We discussed this further in the methods paper (Altvater-Hughes et al., 2024). In the current paper, we include a short discussion to address the points below but also draw comparisons with the percent of sequences that were productive in other studies. Lines 615 to 627

Comment 7: In table 3: blood IgG, they see similar numbers, 60% removal of raw reads and one-third decrease to productive protein. Likewise, table 4: colostral IgM has similar values of 50% and one-third. So at least this is consistent. But when looking at the average SEM from each table, this is much higher in the colostrum compared with blood and this is not discussed.

Author response:

We have added discussion addressing the fact that there is greater variation in the percent of ultralong CDR3s in colostral samples. Lines 585 to 587.

Comment 8: Inconsistencies in data reporting for Cows 1 to 8. When I tried to replicate the data in Figure 5: correlation of IgM ultralongs in colostrum and blood there was no correlation when I used Cows 1 to 7; I could only replicate Figure 5 when I removed Cow 8. Then I noticed a discrepancy in the ‘parity’ column on the tables. In Tables 4 and 5, Cow 1 has a parity of 3 and Cow 8 has a parity of 4. However, in Tables 1 and 3, Cow 1 has a parity of 3 and Cow 8 is not included. In Table 6, Cow 1 has a parity of 4 and Cow 8 has a parity of 1. This is making it impossible to directly compare blood against colostrum for each cow.

Author response:

Thank you for capturing the discrepancy in the tables. The sample without a corresponding blood sample has been removed from the study for improved clarity. Based on rerunning all of the statistics and correlations for the dataset, there is still a tendency for a positive association between the percent of colostral and blood IgM ultralong CDR3 sequences. This discrepancy arose during the addition of the dataset to the SRA and attempting to correspond the appropriate dataset to the tables. Cows are now labelled 1 through 7 in order of decreasing age/parity.

Discussion

Comment 9: Line 590. “There are currently no estimates of the percentage of colostral B cells expressing ultralong CDR3s, but the estimates from this study were higher than what is currently found in the literature regarding the percentage of B cells in blood with ultralong CDR3s.” I am in agreement with this statement, and I suspect part of this may be due to the MACS method of isolating IgG and IgM B cells from blood and the analysis tools used as mentioned above in my comments.

Author response:

Certainly, there is a potential bias inherent in the use of MACs; this is addressed in line 729 to 734. However, the percentages of ultralong CDR3s that are published vary largely in the literature. The variation is likely due to differences in age, study population, primers, and sequencing platforms. Studies in the literature have not used MACs sorting and report lower percentages of ultralong CDR3s in blood than what is reported in the current study for colostrum.

On the other hand, percentages of IgM ultralong CDR3 sequences in colostrum are similar to percentages of blood IgM ultralong CDR3s in mature heifers (Altvater-Hughes et al., 2024) which underwent MACs sorting. The percent of blood IgM ultralong CDR3 sequences from cows at calving are lower than the percent of blood IgM ultralong CDR3 sequences in mature heifers from Altvater-Hughes et al., 2024. There is greater variation in the percent of ultralong CDR3 sequence in colostrum samples than in blood and there may be an influence on calving on the percent of circulating B cells that have an ultralong CDR3.  Lines 585 to 593.

A direct comparison of MACs sorted and samples that were not sorted will need to be completed to understand any bias. However, colostrum is a difficult and viscous sample to work with potentially making sorting through a magnetic column difficult. This is addressed in lines 729 to 734.

From our studies which have not been published yet, age and life stage (heifer, fresh cow, newborn) are factors that drastically influence the percentage of ultralong CDR3s that are in a sample.

Comment 10: Line 612. The discussion would benefit from more-in depth comparison of IMGT versus RSLB analysis.

Author response:

We have included a more in-depth comparison of IMGT and RSLB, which was completed in the study by Altvater-Hughes et al., 2024. We include a brief discussion on the comparison completed in this paper. RSLB filtering method is flexible and transparent and for this reason may be particularly useful for analysis of the bovine antibody repertoire, specific structural motifs, and unique CDR3s. Lines 615 to 627. 

Reviewer 2 Report

Comments and Suggestions for Authors
  1. How did you ensure the purity and viability of the isolated colostral cells after centrifugation, and could the isolation method have affected the comparison between blood and colostrum B cell populations?
  2. You observed a significantly higher percentage of IgM and IgG B cells with ultralong CDR3s in colostrum compared to blood. Could you elaborate on the potential functional significance of these ultralong CDR3s in neonatal immunity? Have any studies previously suggested a specific role for ultralong CDR3s in pathogen defense in neonates?
  3. The sample size for blood (n=7) and colostrum (n=8) appears relatively small. Can you comment on the statistical power of your study? Are the p-values of 0.05 robust enough given the sample size, or might a larger sample be necessary to confirm these findings?
  4. Given the complexity of Nanopore sequencing, particularly with long-read amplicons such as those for ultralong CDR3s, how did you account for sequencing errors or biases that might arise during the amplification and sequencing process? Could this have influenced the observed differences between colostrum and blood samples?
  5. While you report differences in ultralong CDR3 percentages between colostrum and blood, did you explore whether these B cells with ultralong CDR3s in colostrum exhibit functional differences in terms of antigen recognition or pathogen-neutralizing capabilities?

Author Response

Response to Reviewer 2 (Author):

Thank you for your revisions. We have addressed each point in detail below.

Reviewer 2:

Comment 1: How did you ensure the purity and viability of the isolated colostral cells after centrifugation, and could the isolation method have affected the comparison between blood and colostrum B cell populations?

Author response:

After centrifugation of colostral cells, MACs sorting for B cells was not used. The viability of cells was assessed when completing cell counts, but the purity (referring to concentration of B cells) was not assessed, since colostrum is a difficult sample type to work with. Another concern was the limited number of B cells and potentially not capturing the population that was present in colostrum, especially of isotype IgM. This is discussed as a limitation within the discussion and has been expanded from lines 729 to 734.

Comment 2: You observed a significantly higher percentage of IgM and IgG B cells with ultralong CDR3s in colostrum compared to blood. Could you elaborate on the potential functional significance of these ultralong CDR3s in neonatal immunity? Have any studies previously suggested a specific role for ultralong CDR3s in pathogen defense in neonates?

Author response:

There are studies that have investigated colostral derived T cell function in neonatal mammals (Langel et al., 2015 and Reber et al., (2005 to 2008) in the manuscript). To our knowledge, no one has investigated the role of ultralong CDR3s in bovine neonatal health. In essence, this is something that can continue to be investigated based on the foundation of this work. Functionally, bovine ultralong CDR3s seem to have good viral neutralizing functions but this has been investigated in more mature animals and not in the first months of life. Follow up studies about health and protective abilities of ultralong CDR3s would need to be further investigated. In a separate study, we are investigating the repertoire in calves from birth (pre-colostrum) to 10 months of age.

Comment 3: The sample size for blood (n=7) and colostrum (n=8) appears relatively small. Can you comment on the statistical power of your study? Are the p-values of 0.05 robust enough given the sample size, or might a larger sample be necessary to confirm these findings?

Author response:

We acknowledge the sample size is small in non-sequencing studies, but this is the largest study currently for colostral B cells and for long-read sequencing that is investigating ultralong CDR3s in cows. The results are motivation to further expand this study to a larger sample population to include more variables in an analysis. The sample size is addressed as a limitation in the discussion from lines. 719 to 728.

Comment 4: Given the complexity of Nanopore sequencing, particularly with long-read amplicons such as those for ultralong CDR3s, how did you account for sequencing errors or biases that might arise during the amplification and sequencing process? Could this have influenced the observed differences between colostrum and blood samples?

Author response:

Both blood and colostrum were treated the same way from RNA extraction to sequencing so there should be no biases that would exist due to the methods following magnet bead-based cell separation in blood. ONT newest technology has improved to have a sequencing error rate <1%. The same PCR bias would exist among all samples, since they underwent the same protocol. Therefore, PCR and sequencing would not have influenced the difference between samples. The

RSLB filtering method was meant to remove artefacts from sequencing through the manual curation of filtering motifs. Sequence artefacts such as multiple sequences that were read as one or incomplete sequences were discarded using this filtering method. Sequences that did not meet the quality standard were discarded.

Comment 5: While you report differences in ultralong CDR3 percentages between colostrum and blood, did you explore whether these B cells with ultralong CDR3s in colostrum exhibit functional differences in terms of antigen recognition or pathogen-neutralizing capabilities?

Author response:

At this point, functional differences in terms of antigen recognition or neutralizing capabilities have not been explored in the context of this study. Investigation of the functionality is beyond the scope of the current study.